# The influence of vancomycin on renal functions, the predictors and associated factors for nephrotoxicity

**DOI:** 10.1371/journal.pone.0284223

**Published:** 2023-04-17

**Authors:** Waleed M. Altowayan, Mugahid A. Mobark, Abdulmajed ALharbi, Abdullah Ali Alduhami, Syed Imam Rabbani

**Affiliations:** 1 Department of Pharmacy Practice, College of Pharmacy, Qassim University, Buraydah, Saudi Arabia; 2 Department of Pathology, Faculty of Medicine, University of Kordofan, El-Obeid, Sudan; 3 Clinical Pharmacy, King Fahad Specialist Hospital, Buraydah, Qassim, Saudi Arabia; 4 Department of Pharmacology and Toxicology, College of Pharmacy, Qassim University, Buraydah, Saudi Arabia; Pikeville Medical Center, UNITED STATES

## Abstract

**Background:**

Vancomycin has been widely used in the last six decades to treat methicillin-resistant *S*. *aureus* (MRSA) and other resistant gram-positive infections. The risk of vancomycin toxicity increases with the utilization of higher doses while treating the resistant form of bacterial infections. Nephrotoxicity is one of the major complications reported to be a hinderance in the prognosis of vancomycin therapy.

**Objectives:**

This hospital-based study aimed to highlight the influence of vancomycin on renal function with special emphasis on identifying the predictors and augmenting factors for nephrotoxicity.

**Methodology:**

A cross-sectional, unicentric, hospital-based study was conducted at King Fahad Specialist Hospital (KFSH) in Qassim region in Saudi Arabia (KSA). It included 319 hospitalized patients who received vancomycin at intermittent doses (15 to 30 mg/kg IV per day) based on the diseased state. Data regarding vancomycin dose, frequency, duration and data of renal function tests and type of admission were analysed to evaluate their influence on the renal function using parameters such as blood urea, serum creatinine levels and creatinine clearance. One-way ANOVA and Spearman correlation test were used in the analysis of data.

**Results:**

Both male and female patients treated with vancomycin had significantly (p<0.05) elevated blood urea and serum creatinine levels compared to baseline levels while creatinine clearance was non-significantly varied. Increasing age, increasing body weight, higher vancomycin dose and trough levels, increased vancomycin frequency and duration, critically ill patients and site of infection were factors associated with significant (p<0.05) increases in blood urea and serum creatinine levels with reduction in creatinine clearance.

**Conclusion:**

Data suggested that vancomycin treatment reduced the renal function in patients and indicated its association with several predictors and confounding factors. The findings of the study might assist in identifying the patients under risk from the vancomycin-induced nephrotoxicity and in designing the preventive strategies to reduce such complications.

## Introduction

Vancomycin is a glycopeptide antibiotic that has been widely used for over 65 years to treat methicillin-resistant *S*. *aureus* (MRSA) and other resistant gram-positive infections.

In published guidelines, a high dose of vancomycin has been recommended to overcome MRSA strains [1]. However, close monitoring and optimization of vancomycin doses is rather important as sub-dosing contributes to vancomycin resistance while higher extra-dose is associated with risk of toxicity [2]. Most of the therapeutic guidelines recommend monitoring of vancomycin based on the trough concentration [3]. For MRSA infection, the trough recommended concentration is 10 to 20 mg/L, and for severe infection, the recommended concentration is 15 to 20 mg/L [4]. Although vancomycin trough level monitoring is essential for follow-up of patients, other factors that influence vancomycin trough levels need to be considered, as significantly higher vancomycin trough level was seen among female patients and in critically ill patients, in addition to significant correlation with the site of infection [5].

Vancomycin is eliminated from the body primarily by the renal route via both glomerular filtration and active tubular secretion. So in a setting of normal creatinine clearance, vancomycin has a β-elimination half-life of 6–12 hours [6, 7].

The published data related to direct causal association between vancomycin and nephrotoxicity are still limited and further complicated with the presence of multiple confounding nephrotoxic factors, and there is a little evidence of nephrotoxicity with vancomycin used alone [8, 9]. However, vancomycin nephrotoxicity was reported to occur at doses above 4 g/day [10].

According to 2009 vancomycin consensus review, vancomycin-induced nephrotoxicity was defined as “a minimum of two increases in serum creatinine of at least 0.5 mg/dL or a 50% or greater increase in the serum creatinine from baseline after several days of vancomycin therapy” [11]. The reported incidence of vancomycin-associated acute kidney injury (VA-AKI) in hospitalized patients with eGFR > 30 ml/min was 11.7% and serum vancomycin levels of > 20 mg/L; hypotension and administration of iodinated contrast significantly increase this risk [12].

In this retrospective, hospital-based study we investigated the influence of vancomycin on the parameters of renal function within the context of vancomycin dose, frequency, duration and trough levels beside patients’ characteristics, site of infection and the disease status.

## Material and methods

### Study design

This study was a cross-sectional, unicentric, hospital-based study. It was conducted at KFSH in Qassim region in KSA from 1^st^ April 2021 to 30^th^ June 2022. It was approved by the Qassim Region Research Ethics Committee registered at the National Committee of Bio and Med. Ethics (NCBE), numbered: 1441–1064521.

### Study participants

This study included 319 patients diagnosed with bacterial infections who received vancomycin at intermittent doses that varied according to body weight and disease severity, with doses ranging from 15 to 30 mg/kg IV per day. Patients known to have renal impairment and paediatric patients less than 10 years old were excluded from the study. The blood urea, serum creatinine and serum vancomycin trough level assays were run at the hospital central laboratory. For serum vancomycin trough level, the samples were drawn within 15 to 45 minutes before the administration of the fourth vancomycin dose. The creatinine clearance (CrCl) was calculated using Cockcroft-Gault equation [13].

### Data collection

The patients’ data was obtained from KFSH records (laboratory and inpatients’ records). The data collection sheet was designed into three parts; the first part included the demographic characteristics of the patients: age, gender, weight and body mass index. The second part concerned types of patients’ admission (ICU versus non-ICU), the site of infections, and the laboratory results of renal function test: Blood urea, serum creatinine and creatinine clearance. The third part was concerned with vancomycin related data: dose, frequency, duration of treatment and the vancomycin trough level. The mean and standard deviation of the baseline values for renal function test and vancomycin trough level were obtained from the hospital laboratory data based on the stated baseline reference ranges for each test.

### Statistical analysis

Statistical analysis of the results was done using the SPSS IBM 25 software. One-way ANOVA was used to determine the significance variation between treatment and baseline groups. Spearman correlation test was utilized to determine the association between the variables and serum trough level of vancomycin. The confidence interval of 95% (upper and lower) was computed for all the variables tested in the study. P<0.05 was considered to indicate the significance of the results.

## Results

### A. The demographic characters and the effect of vancomycin on the renal function in patients treated with vancomycin

The demographic data of 319 patients treated with vancomycin indicated that males (51.7%) were slightly more than females. Both male and female patients had significantly (P<0.05) elevated blood urea levels compared to baseline levels. The serum creatinine levels were also found to be elevated significantly (P<0.05) in both male and female patients but the creatinine clearance was non-significantly varied compared to baseline values. The trough level analysis indicated that female patients had significantly (P<0.05) higher levels compared to baseline value. In the age-group analysis, patients over 41 years old showed significantly (P<0.05) more vancomycin trough levels and the highest value (above 22 mg/L, P<0.01) was observed in patients older than 81 years. In the age-group, patients aged between 51 and 90 years were found to have significantly (P<0.01) higher blood urea levels compared to baseline. Further, the serum creatinine levels were found to be significantly (P<0.05) elevated in patients aged 10–20 years and 41–90 years when compared to baseline values. Besides, a significantly (P<0.05) lower creatinine clearance was observed in all patients aged above 61 years when comparison was done with baseline value Table 1.

**Table 1:** Effect of vancomycin on the renal function in patients according to demographic characters.

Demographic characters	Frequency (N and %)	Trough level (mg/L)	Renal function		
			Blood urea (m.mol/L)	Serum creatinine (μmol/L)	Creatinine clearance (mL/min)
***Baseline value***	--	12.5 ± 0.83	4.85 ± 0.65	80.00 ± 10.20	115.5 ± 2.15
***Gender***					
Male	165 (51.7)	15.14 ± 0.92	14.36 ± 1.21*	146.01 ± 13.88*	86.33 ± 6.39
Female	154 (48.3)	18.21 ± 1.02*	13.24 ± 1.17*	141.69 ± 15.04*	82.34 ± 7.78
***Age***					
10–20 years	10 (3.1)	11.42 ± 2.66	8.01 ± 3.01	138.46 ± 67.63*	136.44 ± 33.67
21–30 years	36 (11.3)	13.35 ± 1.97	7.87 ± 1.52	124.06 ± 35.67	114.19 ± 12.50
31–40 years	32 (10)	14.15 ± 2.09	10.98 ± 2.27	131.41 ± 35.11	113.69 ± 18.17
41–50 years	43 (13.5)	16.27 ± 1.57*	12.09 ± 2.55	134.14 ± 22.58*	92.22 ± 11.98
51–60 years	53 (16.6)	18.27 ± 2.36*	12.66 ± 1.89**	135.94 ± 27.24*	95.66 ± 14.25
61–70 years	61 (19.1)	19.28 ± 3.78*	16.93 ± 2.51***	163.84 ± 24.70*	59.11 ± 6.37*
71–80 years	42 (13.2)	20.09 ± 2.89*	19.18 ± 2.58**	164.97 ± 31.19*	55.58 ± 12.88*
81–90 years	32 (10)	22.52 ± 3.14**	17.31 ± 2.53*	138.85 ± 4.66*	48.04 ± 6.76*
Above 90 years	10 (3.1)	22.65 ± 3.03**	11.42 ± 1.01	84.01 ± 10.66	47.94 ± 7.41*

Values are expressed as Mean ± S.E.

**Statistics**: One-way ANOVA.

*p < 0.05

**p < 0.01

***p < 0.001 compared with normal.

### B. Effect of vancomycin on renal functions in patients according to their body weight and body mass index

The analysis of vancomycin trough level according to body weight indicated that patients above 71 kgs have significantly (P<0.05) higher levels. Similarly, the patients with BMI greater than 29.1 showed significantly (P<0.05) elevated trough levels compared to baseline.

Further, the analysis of body weight data indicated that patients with weights above 71 kgs and those taking vancomycin had significantly (P<0.05) higher blood urea levels compared to baseline. The level of serum creatinine was found to be significantly (P<0.05) elevated in patients having body weight above 51 kgs compared to baseline. However, no significant difference was observed in the creatinine clearance among patients with different body weights.

The values obtained for body mass index suggested that patients taking vancomycin and having body mass index above 25.1–39.9 have significantly (P<0.05) higher blood urea levels compared to baseline. In patients with BMI above 40, the mean of blood urea (12.35 ± 4.52 mmol/L) is higher than the reference value, but the limited number (6 patients) in this group affects the statistical relationship. Serum creatinine levels were found to be significantly (P<0.05) increased in patients having body mass index above 18.1 compared to baseline. The analysis also indicated that the creatinine clearances were non-significantly affected in all the groups of body mass index patients, except those between 29.1 and 34.9 where it was significantly (P<0.05) low compared to baseline Table 2.

**Table 2:** Effect of vancomycin on renal functions in patients according to their body weight and body mass index.

Variables	Frequency (N and %)	Trough level (mg/L)	Renal function		
			Blood urea (m.mol/L)	Serum creatinine (μmol/L)	Creatinine clearance (mL/min)
***Baseline value***	--	12.5 ± 0.83	4.85 ± 0.65	80.00 ± 10.20	115.5 ± 2.15
***Body weight***					
Less than 50 kgs	29 (9.1)	14.04 ± 2.19	9.61 ± 2.19	83.51 ± 14.34	75.24 ± 11.35
51–70 kgs	141 (44.2)	12.21 ± 2.03	12.37 ± 1.03	151.55 ± 19.87*	86.06 ± 8.47
71–100 kgs	137 (42.9)	16.77 ± 1.38*	15.45 ± 1.13*	143.04 ± 11.60*	80.82 ± 5.91
Above 101 kgs	12 (3.8)	17.08 ± 2.27*	14.61 ± 4.15*	209.01 ± 58.54**	92.21 ± 25.03
***Body mass index***					
Less than 18	8 (2.5)	12.98 ± 2.15	10.24 ± 4.56	91.40 ± 51.73	79.20 ± 19.10
18.1–25	129 (40.4)	12.29 ± 1.36	12.16 ± 1.18	134.50 ± 17.07*	87.61 ± 9.14
25.1–29	90 (28.2)	13.22 ± 1.87	15.03 ± 1.59*	139.72 ± 19.37*	90.78 ± 10.60
29.1–34.9	68 (21.3)	16.41 ± 2.23*	15.43 ± 1.02*	166.42 ± 22.52*	62.52 ± 6.36*
35–39.9	18 (5.6)	17.75 ± 2.20*	15.29 ± 3.42*	144.56 ± 39.73*	88.28 ± 21.30
Above 40	6 (1.9)	17.89 ± 1.29*	12.35 ± 4.52	200.23 ± 86.70**	112.75 ± 47.10

Values are expressed as Mean ± S.E.

**Statistics**: One-way ANOVA.

* *p* < 0.05

** *p* < 0.01 compared with normal.

### C. Effect of vancomycin on the renal functions according to dose, frequency and duration of treatment

Table 3 indicates the influence of vancomycin treatment regimen on the renal function. Patients administered vancomycin above 1250 mg had significantly (P<0.05) higher trough levels compared to baseline. Further, the patients receiving the drug thrice daily indicated elevated (P<0.05) trough levels. And patients administered vancomycin for 14 to 21 days were also found to have significantly (P<0.05) higher trough levels.

**Table 3:** Effect of vancomycin on the renal functions according to dose, frequency and duration of treatment.

Vancomycin regimen	Frequency (N and %)	Trough level (mg/L)	Renal function		
			Blood urea (m.mol/L)	Serum creatinine (μmol/L)	Creatinine clearance (mL/min)
***Baseline value***	--	12.5 ± 0.83	4.85 ± 0.65	80.00 ± 10.20	115.5 ± 2.15
***Dosage***					
500 mg	1 (0.3)	12.87 ± 1.52	12.15 ± 3.52	82.22 ± 8.87	101.52 ± 12.64
750 mg	1 (0.3)	13.92 ± 2.62	16.80 ± 5.91*	104.43 ± 45.74	105.50 ± 27.50
1000 mg	254 (79.6)	14.17 ± 2.56	11.28 ± 1.95	86.57 ± 7.77	114.32 ± 27.20
1250 mg	2 (0.6)	17.11 ± 3.28*	27.92 ± 7.88**	30.08 ± 10.02*	72.33 ± 52.46*
1500 mg	3 (0.9)	17.85 ± 3.67*	48.80 ± 22.50***	22.90 ± 12.60*	76.00 ± 89.00*
***Dose frequency***					
OD	59 (18.5)	12.69 ± 2.11	10.36 ± 4.50	82.26 ± 10.22	99.20 ± 14.53
BID	241 (75.5)	12.92 ± 2.04	10.19 ± 7.87	89.87 ± 9.08	115.05 ± 20.47
EOD	11 (3.4)	13.62 ± 2.35	9.09 ± 3.55	92.58 ± 8.94	118.43 ± 27.14
TID	8 (2.5)	16.82 ± 3.21*	14.63 ± 5.71*	82.29 ± 19.30	71.20 ± 87.54*
***Duration of treatment***					
3 days	22 (6.9)	12.01 ± 1.26	10.02 ± 1.55	82.36 ± 9.81	96.82 ± 9.41
5 days	31 (9.7)	14.84 ± 2.21	11.15 ± 1.44	93.18 ± 14.52	83.05 ± 15.70
7 days	98 (30.7)	13.96 ± 1.97	10.92 ± 2.79	64.76 ± 9.95	94.42 ± 18.09
10 days	18 (5.6)	14.90 ± 1.69	14.22 ± 1.42*	83.74 ± 10.49	71.43 ± 34.26*
14 days	119 (37.3)	17.49 ± 2.38*	13.46 ± 2.65*	90.86 ± 15.42	63.43 ± 20.24*
21 days	29 (9.1)	19.53 ± 2.33**	14.43 ± 5.11*	98.73 ± 31.39	58.25 ± 36.02*
42 days	2 (0.6)	14.46 ± 3.42	7.80 ± 5.20	66.25 ± 24.25	53.29 ± 15.02*

Note: OD–Once daily, BID–Twice a day, EOD–Every other day, TID–Three times a day.

Values are expressed as Mean ± S.E. **Statistics**: One-way ANOVA.

*p < 0.05

**p < 0.01

***p < 0.001 compared with normal.

The doses of vancomycin such as 750 mg (P<0.05), 1250 mg (P<0.01) and 1500 mg (P<0.001) were found to significantly increase the blood urea levels compared to baseline. Further, two tested doses of vancomycin (1250 mg and 1500 mg) significantly (P<0.05) increased the serum creatinine levels and also reduced the creatinine clearance compared to baseline values.

Dose frequency of vancomycin suggests that when the drug was administered three times a day, the blood urea level was found to be significantly (P<0.05) increased compared to baseline. Similarly, this frequency of vancomycin dosing also significantly reduced (P<0.05) the creatinine clearance compared to baseline. The duration of treatment data reveal that vancomycin administration for 10–21 days is associated with significant (P<0.05) elevation of blood urea levels compared to baseline. And more than 10 days of vancomycin treatment significantly (P<0.05) reduced the creatinine clearance compared to control values.

### D. Effect of vancomycin on renal function in different types of patients’ admission and site of infections

Analysis of the data suggested that patients admitted to ICU and CSICU have significantly (P<0.05) high vancomycin trough levels compared to baseline. Basically, the patients having infections of blood, abdomen, heart, sepsis, urinary tract and wounds indicated significantly (P<0.05) elevated vancomycin trough levels.

Depending on patients’ admission, the analysis of data also suggested that ICU (P<0.05) and CSICU (P<0.01) patients receiving vancomycin have significantly (P<0.05) higher blood urea levels. These admissions also had elevated serum creatinine levels [ICU (P<0.05) and CSICU–(P<0.01)] when compared with baseline values. The creatinine clearance was found to be significantly reduced in patients admitted to CCU (P<0.05), ICU (P<0.05) and CSICU (P<0.001) wards.

Influence of site of infection on kidney function suggested that infection of blood, heart, sepsis risk, urinary tract and lungs with vancomycin treatment had significantly (P<0.05) higher blood urea levels compared to baseline. Vancomycin treatment in patients diagnosed with infection of abdomen, heart, sepsis risk, urinary tract, wound and lungs showed significantly (P<0.05) higher serum creatinine levels. However, the creatinine clearance was found to be significantly (P<0.05) low only in patients indicated with heart and wound infections Table 4.

**Table 4:** Effect of vancomycin on renal function in different types of patients’ admission and site of infections.

Type of patients’ admission	Frequency (N and %)	Trough level (mg/L)	Renal function		
			Blood urea (m.mol/L)	Serum creatinine (μmol/L)	Creatinine clearance (mL/min)
***Baseline value***	--	12.5 ± 0.83	4.85 ± 0.65	80.00 ± 10.20	115.5 ± 2.15
***Patients’ admission***					
Non-emergency	124 (38.8)	12.59 ± 1.88	12.46 ± 2.11	78.95 ± 5.64	99.24 ± 6.61
CCU	22 (6.9)	14.26 ± 4.51	11.94 ± 1.68	126.79 ± 17.35	61.86 ± 10.87*
ICU	171 (53.6)	16.43 ± 2.67*	16.07 ± 1.48*	151.27 ± 17.48*	70.14 ± 5.54*
CSICU	2 (0.6)	17.01 ± 3.16*	23.30 ± 15.30**	192.66 ± 19.02**	39.25 ± 3.55***
***Site of infection***					
Blood	121 (37.9)	16.26 ± 2.37*	15.25 ± 8.84*	118.12 ± 9.64	71.96 ± 5.97
Abdomen	4 (1.2)	17.16 ± 3.03*	11.02 ± 6.51	143.60 ± 99.67*	116.30 ± 54.08
Brain	14 (4.3)	13.21 ± 1.82	6.88 ± 1.31	87.1 ± 7.74	101.75 ± 5.21
Heart	10 (3.1)	18.01 ± 3.76*	14.29 ± 4.28*	131.71 ± 33.41*	55.36 ± 12.33*
Sepsis risk	12 (3.8)	16.81 ± 2.31*	14.65 ± 4.67*	144.25 ± 54.94*	91.14 ± 25.93
Urinary tract	17 (5.3)	17.12 ± 3.18*	22.51 ± 6.37**	181.88 ± 64.93*	87.51 ± 36.63
Wounds	12 (3.7)	16.79 ± 2.46*	12.44 ± 4.13	223.25 ± 132.65**	47.37 ± 12.84*
Lungs	98 (30.7)	13.64 ± 3.07	13.27 ± 1.29*	125.05 ± 19.78	82.73 ± 8.67

Note: CCU–Critical care unit, ICU–Intensive care unit, CSICU–Cardiac surgery intensive care unit.

Values are expressed as Mean ± S.E. **Statistics**: One-way ANOVA.

*p < 0.05

**p < 0.01

***p < 0.001 compared with normal.

### E. Correlation summary between the variables and renal functions after treatment with vancomycin

The comparative analysis between groups indicated that age (Rho = 0.342), duration of treatment (Rho = 0.273), patients’ admission (Rho = 0.321) and site of infection (Rho = 0.406) have significantly (P<0.05) higher vancomycin trough levels. Further, the correlation analysis done to determine the association between the confounding factor and outcome suggested that age, dose, frequency of dosing, patient’s admission and site of infection have significantly (P<0.05) influenced the elevated blood urea levels in patients receiving the vancomycin. The relationship was found to be more (Rho = 0.448) for the site of infection. Body weight, patient’s admission and site of infection were found to have a significant (P<0.05) role in the elevated serum creatinine levels. On the other hand, body mass index, dose and patient’s admission significantly (P<0.05) influenced the lower creatinine clearance in patients receiving the vancomycin treatment. The relationship was observed to be more significant (Rho = 0.385) between the increasing dose of vancomycin and low creatinine clearance Table 5.

**Table 5:** Correlation summary between the variables and renal functions after treatment with vancomycin.

Variables	Rho value of Spearman correlation (Lower and upper 95% CI)			
	Trough level	Blood urea	Serum creatinine	Creatinine clearance
Gender	0.012 (0.008–0.019)	0.014 (0.008–0.021)	0.068 (0.033–0.082)	0.036 (0.011–0.082)
Age	0.342* (0.155–0.401)	0.211* (0.167–0.280)	0.026 (0.019–0.045)	0.009 (0.002–0.014)
Body weight	0.009 (0.006–0.011)	0.007 (0.004–0.012)	0.205* (0.177–0.269)	0.012 (0.008–0.031)
BMI	0.135 (0.008–0.215)	0.024 (0.011–0.063)	0.077 (0.045–0.091)	0.266* (0.191–0.306)
Dose	0.103 (0.007–0.159)	0.146* (0.098–0.171)	0.052 (0.029–0.073)	0.385** (0.261–0.412)
Frequency of dosage	0.181 (0.168–0.269)	0.192* (0.081–0.212)	0.041 (0.036–0.061)	0.016 (0.008–0.025)
Duration of treatment	0.273* (0.169–0.312)	0.046 (0.022–0.086)	0.083 (0.055–0.116)	0.092 (0.062–0.130)
Patient’s admission	0.321* (0.298–0.359)	0.251* (0.198–0.302)	0.125* (0.079–0.154)	0.225* (0.191–0.278)
Site of infection	0.406** (0.378–0.468)	0.448** (0.376–0.511)	0.298* (0.204–0.327)	0.068 (0.021–0.082)

**Statistics**: Spearman correlation test. *P-*value indicated comparison between groups

(*p < 0.05

**p < 0.01)

## Discussion

Although Vancomycin-associated nephrotoxicity has been faced with initial doubts as multiple other confounding risk factors may exist, more than one study reported an increased risk of nephrotoxicity with higher doses of vancomycin [1, 10, 14]. In this cross-sectional, unicentric, hospital-based study that was conducted at KFSH among 319 patients treated with vancomycin, the findings showed that both male and female patients treated with vancomycin had significant (*P*<0.05) elevated blood urea and serum creatinine levels compared to baseline levels. In 2013, a systematic review and meta-analysis study found that the collective literature reports a relationship between vancomycin exposure and nephrotoxicity and the causal association is more expected with increased vancomycin trough level concentration and the duration of therapy [15].

In this current study, we calculated the CrCl using Cockcroft-Gault formula and the findings in both male and female showed non-significant variation compared to baseline values. However, the effect of variation in body weight among the study participants should be considered. When using Cockcroft-Gault equation, overestimation of CrCl is expected especially for obese patients as their TBW includes a disproportionately greater amount of non-muscle tissue as compared to the Cockcroft and Gault study population [16]. Interestingly, a meta-analysis of 13 studies done by Wilhelm and Kale-Pradhan reported that “using TBW in Cockcroft-Gault equation overestimates measured CrCl and using IBW underestimates measured CrCl, while using an adjusted body weight (ABW) with a correction factor of either 0.3 or 0.4 to adjust for the difference between TBW and IBW produced a slight overestimation” [17]. In another study, Aimo Harmoinen and his colleagues reported that GFR values calculated by the Cockcroft-Gault formula were ∼10% higher than the plasma 51Cr-EDTA clearance values [18]. Shoker and his colleagues suggested that “normalization for body surface area in the original Cockcroft-Gault formula demonstrated more accuracy to estimate creatinine clearance” [19].

This study found a significant increase in vancomycin trough levels compared to baseline in patients above 41 years and, linked to that, significantly higher blood urea levels compared to baseline were found in patients aged between 51 and 90 years. Further, the serum creatinine levels were found to be significantly elevated in patients 41–90 years when compared to baseline values. In addition to that, a significant (P<0.05) lower creatinine clearance in all patients aged above 61 years is seen.

As reported previously, the risk of vancomycin-associated nephrotoxicity was higher among elderly patients compared to younger patients [20]. Moreover, the risk of vancomycin-associated nephrotoxicity increases to 3-fold with vancomycin trough concentrations of >15 mg/ml [21].

In the present study the analysis of vancomycin trough level indicated significantly higher levels in patients who weighed over 71 kgs and BMI greater than 29.1. Similarly, higher blood urea levels compared to baseline were found in patients who weighed more than 71 kgs while serum creatinine was found to be significantly elevated in patients weighing over 51 kgs. Regarding BMI, significantly higher blood urea levels compared to baseline were found in patients having body mass index above 25.1–39.9. The analysis also indicated that the creatinine clearances were non-significantly affected in all the groups of body mass index patients, except 29.1–34.9 where it was significantly (P<0.05) low compared to baseline. These findings also point to the effect of weight on calculation of creatinine clearance using Cockcroft-Gault formula. The increase in body weight increases the drug exposure based on dosing calculations and volume of distribution estimation. In line with these findings, in a review study from 2016 they reported obesity as a risk factor for vancomycin-associated nephrotoxicity [22]. More than one study also reported obesity as a risk for vancomycin-associated nephrotoxicity [23–25]. In contradiction to these findings, a study done in 2015 for obesity as a factor in vancomycin-associated nephrotoxicity concluded that “no difference in nephrotoxicity was observed between lean and obese patients treated with vancomycin at our institution” [9].

This study indicated that vancomycin dose above 1250 mg, frequency of thrice daily and duration of 14 to 21 days had significantly higher trough levels compared to baseline. Linear to these, the doses of vancomycin such as 750 mg, 1250 mg and 1500 mg, and vancomycin administration three times a day significantly increased the blood urea and serum creatinine levels and reduced the creatinine clearance compared to baseline values. The vancomycin administration for 10–21 days is associated with significant elevation of blood urea levels and reduction in creatinine clearance. The increase in dose, frequency and duration of vancomycin logically increases the drug exposure reflected as increased vancomycin trough levels and hastens the mechanism by which vancomycin affects renal function. The risk of vancomycin-associated nephrotoxicity with the use of large doses was reported in many studies [10, 26–29]. In another study, Contreiras and his colleagues concluded that “patients receiving vancomycin for more than 7 days had an increased likelihood of experiencing nephrotoxicity” [30].

The current study highlighted that ICU and CSICU admitted patients have significantly high vancomycin trough levels compared to baseline together with significantly higher blood urea, higher serum creatinine levels and significantly reduced creatinine clearance. Besides that, the patients’ having infections of blood, abdomen, heart, sepsis, urinary tract and wound indicated significantly elevated vancomycin trough levels and significant elevation of both blood urea and serum creatinine. The critically ill patients are hemodynamically unstable and kidney function is already compromised with the underlying disease state so they are at greater risk of renal deterioration following use of medications even with minimal risk of injury to the kidneys. Moreover, critically ill patients receive multiple medications which further increases the risk of nephrotoxicity. Vancomycin dose adjustment, close monitoring and observation of renal function is of utmost importance in critically ill patients. Malacarne and colleagues reported a significant nephrotoxic event occurred in critically ill patients with septic shock due to concurrent administration of vancomycin and aminoglycosides [31]. In critically ill patients with cardiac surgery, vancomycin was reported to have an association with deterioration of renal function [32]. Most of the reported vancomycin-associated nephrotoxicity occurs in critically ill patients [22, 33–35].

## Conclusion

This cross-sectional, unicentric, hospital-based study at KFSH found that both male and female patients treated with vancomycin had significantly elevated blood urea and serum creatinine levels compared to baseline levels while CrCl calculated by Cockcroft-Gault formula showed non-significant variation compared to baseline values. Increased age, increase in weight, higher vancomycin dose, increased frequency, prolonged duration of treatment, critically ill patients and site of infection have significantly higher vancomycin trough levels and significantly elevated blood urea and serum creatinine levels compared to baseline with significantly influenced lower creatinine clearance. These factors can be evaluated and regarded as pre-treatment predictors of vancomycin-associated nephrotoxicity, especially in critically ill patients.

### Limitations of the study

- The primary limitation of this study is the retrospective design with a limited number of patients.
- The inherent factors and the state of the infection may have an impact on the renal function and represent confounders for accurate judgment regarding vancomycin-associated nephrotoxicity.
- History of long term use of medications is not addressed and certain medications have a negative impact on renal function.
- Calculation of CrCl using Cockcroft-Gault formula as measured creatinine clearance would yield more accurate results.
